# Time-restricted feeding induces *Lactobacillus*- and *Akkermansia*-specific functional changes in the rat fecal microbiota

**DOI:** 10.1038/s41522-021-00256-x

**Published:** 2021-12-03

**Authors:** Antonio Palomba, Alessandro Tanca, Marcello Abbondio, Rosangela Sau, Monica Serra, Fabio Marongiu, Cristina Fraumene, Daniela Pagnozzi, Ezio Laconi, Sergio Uzzau

**Affiliations:** 1 Porto Conte Ricerche Srl, Science and Technology Park of Sardinia, Alghero, Italy; 2 Department of Biomedical Sciences, University of Sassari, Sassari, Italy; 3 Department of Biomedical Sciences, University of Cagliari, Cagliari, Italy

**Keywords:** Microbiome, Applied microbiology

## Abstract

Diet is a key factor influencing gut microbiota (GM) composition and functions, which in turn affect host health. Among dietary regimens, time-restricted (TR) feeding has been associated to numerous health benefits. The impact of TR feeding on the GM composition has been mostly explored by means of metagenomic sequencing. To date, however, little is known about the modulation of GM functions by this dietary regimen. Here, we analyzed the effects of TR feeding on GM functions by evaluating protein expression changes in a rat model through a metaproteomic approach. We observed that TR feeding has a relevant impact on GM functions, specifically leading to an increased abundance of several enzymes involved in carbohydrate and protein metabolism and expressed by *Lactobacillus* spp. and *Akkermansia muciniphila*. Taken together, these results contribute to deepening our knowledge about the key relationship between diet, GM, and health.

## Introduction

Lifestyle interventions, including changes in diet and increased exercise, result in many health benefits able to prevent (and enhance treatment of) various metabolic diseases. Among dietary interventions, caloric restriction (CR) has been most intensely investigated. CR consists in a reduction of total daily calories intake without changing the macronutrient composition nor causing malnutrition. Studies in animals and humans have repeatedly reported that CR leads to an extension of both lifespan and healthspan^1,2^. However, the applicability of the CR regimen is limited, due to the difficulties to be maintained over a long time and to be used in the management of chronic conditions^3^.

An alternative dietary approach, that is being actively explored for its potential benefits, is intermittent fasting (IF). IF regimens were studied both in human subjects and animal models and proved to ameliorate a variety of pathological conditions, including obesity, impaired glucose tolerance, dyslipidemia, hypertension, fertility problems, liver impairment, and neurodegenerative diseases^4,5^. IF has been recently categorized in whole-day fasting, every other day fasting, and time-restricted (TR) feeding^6^. In the TR feeding protocol, food consumption is not randomly distributed across the 24 h, but it is limited to a daily interval of 3–4^7^, 7–9^8^, or 10–12 h^9^, allowing daily fasting duration greater than 12 h. A basic rationale of this dietary regimen is that the feeding time period should be aligned with internal circadian rhythms, in order to synchronize with the active phase of animal or human metabolism^10,11^. The benefits of this feeding regimen appear to be proportional to fasting duration^6^. It is important to note that during the TR feeding regimen the quality and quantity of nutrients is comparable to that of *ad libitum* (AL) controls. In fact, after a short training period (usually lasting less than one week) under controlled experimental conditions, animals exposed to TR feeding are able to eat ≥90% of the food ration consumed by AL-fed controls^12^. TR feeding, unlike CR, can be well tolerated by humans for long periods, also at repeated intervals, as noted in the Islamic fasting during the month of Ramadan^13^. An increasing number of studies have indicated that TR can reproduce at least some of the effects associated with CR^14^, including stimulation of autophagy, increased mitochondrial respiratory efficiency, modulation of reactive oxygen species, and changes in the profile of inflammatory cytokines^15^. Furthermore, TR was shown to reduce body fat mass^16^, to decrease plasma levels of triglycerides and low-density lipoproteins^6,17^, and to increase lifespan in mice^18^.

Diet is a key factor influencing gut microbiota (GM) composition and functions, which in turn affect host health^19^. It is well-known that GM composition varies in response to isocaloric diets differing in macronutrient composition, as well as to diets with identical macronutrient composition differing in caloric content^20^. Recently, a few studies specifically investigated the possible impact of TR feeding on GM composition. Ren et al. found alterations in the GM caused by TR feeding linked to recovery from hepatic ischemia-reperfusion injury^21^. Zeb et al. reported that a TR diet can impact the GM, influencing in turn host metabolism and nutritional status^22^. The same research group also showed that TR feeding can enhance GM richness (specifically Prevotellaceae and Bacteroidaceae diversity). Hu et al. demonstrated that a TR feeding regimen in juvenile mice has long-term effects on the GM, leading to a disturbed microbiota-host relationship that can hardly be solved in later life^23,24^, suggesting that TR-derived effects might be influenced by the age at which the dietary changes occur.

GM metabolic processes exert a significant impact on the host physiology, affecting gut mucosa homeostasis and being key to the dynamic reciprocal relationship established between the gut and other host systems. In this respect, metaproteomics (unlike metagenomics) can provide reliable information on which biological processes are actually activated (or repressed) by the GM in response to host or environmental stimuli^25^, by measuring variations occurring in microbial protein abundance and in the corresponding biochemical pathways. Furthermore, metaproteomics can infer which members of the GM are involved in specific molecular functions. However, no studies to date have investigated the influence of TR feeding on GM through a metaproteomic approach.

In the present study, we aimed to investigate the effects of long-term TR feeding on GM protein expression in a rat model, by exploiting an established fecal metaproteomic approach allowing a taxon-specific functional characterization of the GM. In parallel, samples were analyzed by 16S rRNA gene sequencing, to provide a gold-standard evaluation of GM taxonomic structure. Taxon-specific processes and pathways possibly influenced by TR feeding were examined and discussed.

## Results

### Experimental design and general metrics

**Fig. 1:**
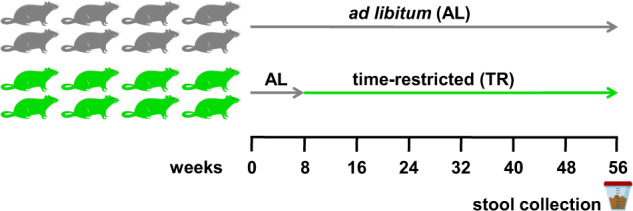
Experimental design. Schematic representation of the experimental design of the study.

Figure 1 shows the experimental design of the study. After 8 weeks of AL feeding, 16 rats were divided into two groups of 8 rats each: the first group was kept on an AL regimen, whereas the second shifted to TR feeding. Food intake and body weight curves regarding an experiment based on the same dietary regimens and carried out on the same type of rats have been previously published^12^.

After 48 weeks of dietary regimen, stool samples were collected from each rat and divided into two fractions. The first stool fraction was subjected to DNA extraction and 16S rRNA gene amplification and sequencing, for a preliminary investigation of GM taxonomic composition through a widely established approach. The second stool fraction underwent protein extraction and filter-aided sample preparation (FASP) protocol for clean-up, alkylation, and digestion; the so obtained peptide mixtures were then analyzed by liquid chromatography coupled with high-resolution mass spectrometry, according to a shotgun metaproteomics approach, to carry out a functional characterization of the GM.

Concerning 16S rRNA gene sequencing data (for details see Supplementary Data 1), a total of 961,072 reads were obtained from rat fecal samples (60,067 on the average per sample), corresponding to 1510 amplicon sequence variants (ASVs). These, in turn, were taxonomically assigned to 51 families, 88 genera, and 43 species (of which 25 with complete binomial nomenclature), as described in Supplementary Data 2.

Concerning metaproteomic data (for details see Supplementary Data 3), we selected four different sequence databases for peptide search (see Methods for further details), namely two collections of rat metagenomic sequences (DB1, containing in-house generated sequences, and DB2, a publicly available database), a rat reference proteome (DB3) and a food database (DB4). A total of 37,341 different peptide sequences were identified and quantified (21,300 on average per sample), distributed as follows among the four databases: 41% in common between DB1 and DB2, 29.5% exclusively from DB1, 20.5% exclusively from DB2, 7.5% from DB3 and 1.5% from DB4. Initially, we aimed at identifying the best performing approaches for taxonomic and functional annotation of the rat metaproteome, as shown in Supplementary Data 4. First, we compared the performance of Unipept (tryptic peptide-, UniProt-based tool) and MEGAN (protein BLAST-, NCBI-based tool) regarding taxonomic annotation. Annotation yields reached by MEGAN were almost double than those reached by Unipept at all levels (e.g., 28.4% vs. 12.8% of peptide sequences annotated at the genus level), although Unipept led to much higher richness levels (e.g., 312 vs. 39 different taxonomic genera detected in the study). Based on the better annotation yield, we chose MEGAN as a taxonomic annotation tool for this study. According to the MEGAN taxonomic classification, 30,331 peptide sequences (17,331 on the average per sample) were assigned to microbial taxa, while 2709 peptide sequences (1695 on the average per sample, corresponding to 527 proteins) were assigned to the host (rat).

Then, we compared the performance of two functional annotation approaches: (i) alignment of protein sequences against a Swiss-Prot database and retrieval of “protein family” information from UniProt, and (ii) processing of protein sequences with the eggNOG-mapper tool and retrieval of “KEGG Orthology Groups” (KOGs). The latter approach provided the best performance, with 69% of peptide sequences annotated vs. 57% with the former approach. Therefore, KOGs were chosen as the main category for functional annotation, with 1387 KOGs detected in total in the study. Other levels of functional annotation, such as the more generic “Cluster of Orthologous Groups” (COGs) and the more specific “Carbohydrate-Active enzymes” (CAZy) and “metabolic pathways”, were also used in various points of this study.

Statistics of combined taxonomic-functional annotations are provided in Supplementary Data 5.

### Taxonomic changes induced by TR feeding in the rat fecal microbiota based on 16S rRNA gene sequencing

**Fig. 2:**
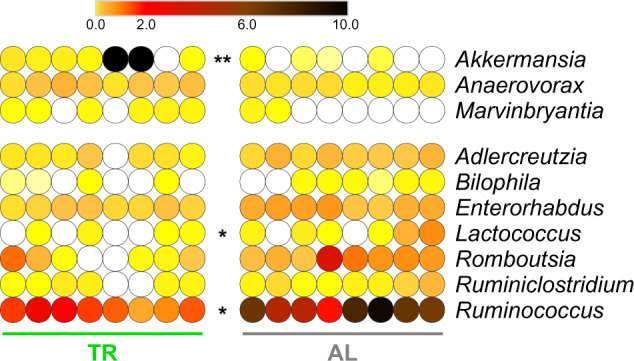
Changes in taxonomic genus composition in the fecal microbiota of TR- vs. AL-fed rats according to 16S rRNA gene sequencing results. Heatmap illustrating genera with significantly differential abundance between AL and TR groups (FDR < 0.1). A single or double asterisk refers to FDR < 0.01 or <0.001, respectively. Each dot indicates a different sample. The color gradient is based on the relative abundance of the genus.

To verify whether TR diet could affect the structure of the rat GM, we investigated its taxonomic composition through 16S rRNA gene sequencing. After a preliminary unsupervised data evaluation through Principal Coordinate Analysis (PCoA; Supplementary Fig. 1), we compared the GM structures of TR- and AL-fed rats with the aim of identifying differentially abundant families (Supplementary Fig. 2), genera (Fig. 2) and species (Supplementary Fig. 3). We found the lineage Akkermansiaceae/*Akkermansia/A. muciniphila*, as well as Prevotellaceae (among families), *Anaerovorax* and *Marvinbryantia* (among genera) and *Ruminococcus flavefaciens* (among species, although the corresponding genus went in the opposite direction, as stated below), among taxa enriched in the GM of TR-fed compared to AL-fed rats. On the other hand, several taxa resulted higher in AL-fed compared to TR-fed rats, such as Atopobiaceae, Erysipelotrichaceae and Ruminococcaceae among families, *Adlercreutzia*, *Enterorhabdus*, *Bilophila*, *Lactococcus*, *Romboutsia*, *Ruminiclostridium*, and *Ruminococcus* among genera, as well as two species belonging to the genus *Bacteroides* (namely, *B. rodentium* and *B. thetaiotaomicron*). Complete results of differential analysis based on 16S rRNA gene sequencing data are shown in Supplementary Data 6.

### Taxonomic changes induced by TR feeding in the rat fecal microbiota based on metaproteomic data

**Fig. 3:**
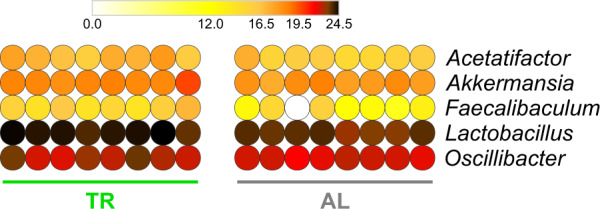
Changes in taxonomic genus composition in the fecal metaproteome of TR- vs. AL-fed rats. Heatmap illustrating microbial genera with significantly differential abundance between AL and TR groups (FDR < 0.1). Each dot indicates a different sample. The color gradient is based on the relative abundance of the genus.

The fecal samples analyzed by 16S rRNA gene sequencing were further investigated to characterize the rat GM metaproteome. Initially, we focused on the taxonomic data, performing a preliminary unsupervised data evaluation via PCoA (Supplementary Fig. 4) and then a differential analysis between the two experimental groups at the family (Supplementary Fig. 5), genus (Fig. 3), and species (Supplementary Fig. 6) level. Among taxa enriched in the GM of TR-fed compared to AL-fed rats, we found again *Akkermansia/A. muciniphila*, together with Lactobacillaceae/*Lactobacillus/L. reuteri*, Oscillospiraceae/*Oscillibacter*, Desulfovibrionaceae, *Acetatifactor*/*A. muris*, *Faecalibaculum*/*F. rodentium* and *Treponema succinifaciens*. The only taxon being more abundant in AL-fed compared to TR-fed rats was *Eubacterium plexicaudatum*. Complete results of differential analysis based on metaproteomic taxonomic data are shown in Supplementary Data 7.

### Taxon-specific functional changes induced by TR feeding in the rat fecal microbiota

**Fig. 4:**
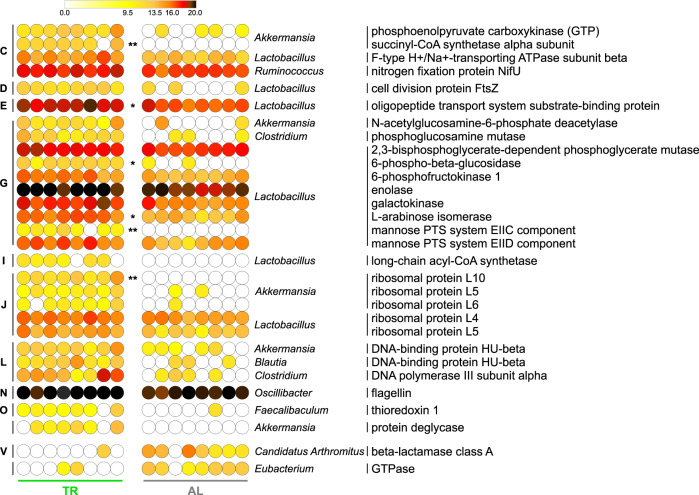
Changes in the functional profile of the fecal metaproteome of TR- vs. AL-fed rats. Heatmap illustrating genus-specific functions with significantly differential abundance between AL and TR groups (FDR < 0.1). A single or double asterisk refers to FDR < 0.01 or <0.001, respectively. Each dot indicates a different sample; the color gradient is based on the relative abundance of functions. Functions are ordered sequentially: (i) according to the group of rats in which they are significantly more abundant, indicated in the bottom; (ii) according to their COG category, indicated close to the left margin of the heatmap (C, Energy production and conversion; D, Cell cycle control, cell division, chromosome partitioning; E, Amino acid transport and metabolism; G, Carbohydrate transport and metabolism; I, Lipid transport and metabolism; J, Translation, ribosomal structure and biogenesis; L, Replication, recombination and repair; N, Cell motility; O, Posttranslational modification, protein turnover, chaperones; V, Defense mechanisms); (iii) according to the taxonomic genus to which they were assigned, indicated close to the right margin of the heatmap; (iv) in alphabetical order.

Next, we focused on the key output of metaproteomics, i.e., the functional analysis of the GM. Specifically, we combined functional (KOG) and taxonomic (family, genus, or species) information assigned to each peptide sequence. After the PCoA unsupervised evaluation (Supplementary Fig. 7), a differential analysis between the two experimental groups was performed, which led to the identification of numerous taxon-specific differential functions, listed in Supplementary Fig. 8 (family-specific functions), Fig. 4 (genus-specific functions), and Supplementary Fig. 9 (species-specific functions). As shown in the Figures, most of the differential functions were expressed by mucus colonizers *Lactobacillus* and *Akkermansia* members.

Specifically, numerous *Lactobacillus*/Lactobacillaceae enzymes were detected as significantly more abundant in the GM of TR-fed rats, involved in glycolysis (phosphoglycerate mutase, enolase, 6-phosphofructokinase), galactose metabolism (galactokinase, alpha-galactosidase), pentose and glucuronate interconversions (arabinose isomerase), fatty acid oxidation (long-chain acyl-CoA synthetase) and glycan degradation (6-phospho-beta-glucosidase, belonging to the glycosyltransferase family GT1). Moreover, the list of microbial functions induced by TR feeding included proteins involved in cell division (cell division protein FtsZ), carbohydrate transport (mannose PTS system components), and peptide transport (oligopeptide transport system substrate-binding protein).

Furthermore, the following protein functions are expressed by Akkermansiaceae/*Akkermansia/A. muciniphila* were found higher in TR-fed rats: several enzymes, including succinyl-CoA synthetase (TCA cycle), phosphoenolpyruvate carboxykinase (gluconeogenesis), N-acetylglucosamine-6-phosphate deacetylase (aminosugars metabolism/peptidoglycan recycling), and protein deglycase (protein/nucleotide repair), as well as histone-like (DNA-binding protein HU-beta) and ribosomal proteins.

Interestingly, the only two functions higher in AL-fed rats were a beta-lactamase class A from *Candidatus* *Arthromitus* and a GTPase from *Eubacterium*. Noteworthy, no significant differences in abundance could be found concerning host (rat) protein functions.

Aggregating data at a more general annotation level, namely “metabolic pathway”, we found that several *Lactobacillus*-specific pathways, including l-arabinose degradation via l-ribulose, galactose metabolism and peptidoglycan biosynthesis, resulted as significantly more abundant in TR-fed rats.

Complete results of differential analysis based on metaproteomic functional and combined taxonomic-functional data are shown in Supplementary Data 8 and 9, respectively.

### Proteomic profiles of the main fecal microbiota members in rats subjected to TR feeding

**Fig. 5:**
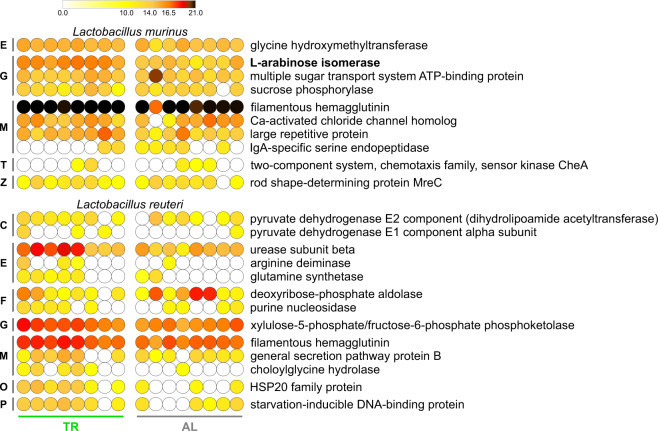
*Lactobacillus murinus*/*reuteri*-specific functions detected in fecal metaproteome samples of TR- and AL-fed rats. Functions in bold are differentially abundant between the two groups (FDR < 0.1; see Supplementary Fig. 9). Each dot indicates a different sample. The color gradient is based on the relative abundance of functions. Functions are ordered sequentially: (i) according to their COG category, indicated close to the left margin of the heatmap (C, Energy production and conversion; E, Amino acid transport and metabolism; F, Nucleotide transport and metabolism; G, Carbohydrate transport and metabolism; M, Cell wall/membrane/envelope biogenesis; O, Posttranslational modification, protein turnover, chaperones; P, Inorganic ion transport and metabolism; T, Signal transduction mechanisms; Z, Cytoskeleton); (ii) according to their average relative abundance.

To gain insight into the contribution of specific members to the functional activity of the GM in TR- and AL-fed rats, we investigated the proteome profile expressed by the two most represented *Lactobacillus* species, namely *L. murinus* and *L. reuteri*. As shown in Fig. 5, the two species-specific functional patterns differed considerably, with filamentous hemagglutinin being the only common protein, *L. murinus* being more involved in carbohydrate transport and metabolism, and *L. reuteri* mostly exerting functions related to amino acid and nucleotide metabolism. To have a broader picture, the complete *Lactobacillus* proteome (genus-specific functions detected in all the samples) is presented in Supplementary Fig. 10, with differentially abundant functions in bold (in common with Fig. 4).

Furthermore, *Akkermansia*-specific functions are listed in Supplementary Fig. 11. Of note is the presence of enzymes involved in aminosugar metabolism, citric acid cycle, and response to oxidative stress.

### TR feeding promotes the expression of galactose metabolism enzymes in *Lactobacillus*

**Fig. 6:**
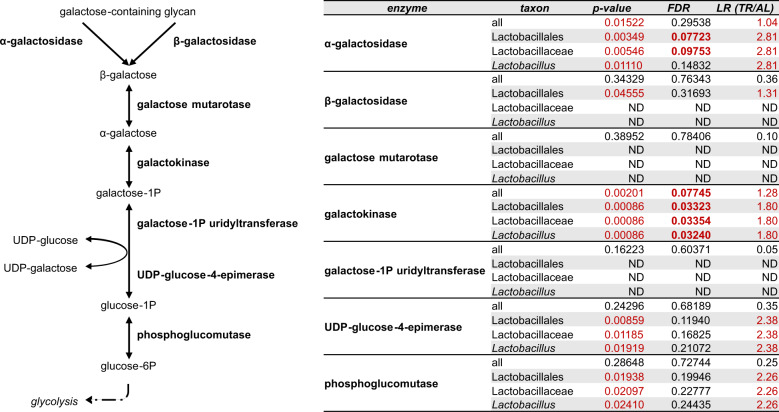
Leloir pathway, galactose metabolism, and lactobacilli in the fecal microbiota of TR- vs. AL-fed rats. Left, schematic representation galactose metabolism enzymes (bold) and metabolites, with focus on the Leloir pathway. Right, taxonomic annotation of Leloir pathway/galactose metabolism enzymes detected in the samples analyzed in this study. *p*-value, FDR, and log-ratio (LR) value obtained upon TR vs. AL differential analysis are reported for each enzyme-taxon combination. *p*-values < 0.05, FDRs < 0.1 and LRs > 1 are shown in red; FDR values < 0.1 are also in bold. ND, not detected (or not passing the filters).

Examining enzymatic functions showing abundance changes associated with TR feeding and assigned to lactobacilli, we noticed that a group of them belong to the galactose metabolism pathway. Therefore, we decided to inspect the abundance data of all enzymes potentially involved in the degradation and biosynthesis of galactose-containing glycans, with a special focus on the Leloir pathway (as shown in Fig. 6).

Interestingly, all enzymes converging on this pathway, identified in this study and attributable to lactobacilli (taxonomic levels from order Lactobacillales down to genus *Lactobacillus* are presented), exhibited a higher mean abundance in the metaproteome of TR-fed rats together with a non-adjusted *p*-value < 0.05, even if the false-discovery rate (FDR) threshold (0.1) was passed in only two cases. This suggests the existence of a differential galactose metabolism-related trend induced by TR feeding.

## Discussion

Diet is known as one of the most important factors influencing GM. Recently, some research groups have explored the impact of TR feeding on GM structure, but no information is available about its effect on GM functions. Fasting, per se, is associated with increased gastrointestinal transit^26^. When fasting is extended for up to 12 h, gastrointestinal transit of ingested food is expected to be completed before the intake of new food^27^. Hence, the small intestine, cecum, and proximal colon are expected to be relatively free of dietary nutrients for about 12 h a day. Endogenous substrates available for microbes colonizing the distal ileum, cecum, and colon are mainly derived from host secreted proteins and peptides, exfoliated cells, and mucins (of salivary, gastric, bronchial, hepato-biliary, small intestinal, and colonic origin). While secretions from the pancreas and other glands are stimulated by the entry of the acidic chyme into the duodenum, mucins are produced continuously and represent a continuous source of proteins and carbohydrates for the “fasting” GM. In this scenario, although challenging, metaproteomic analyses have the potential for a more complete understanding of the GM ecosystem than sequencing alone. Metaproteome data include both taxonomic and functional annotations of proteins, enabling the characterization of the main metabolic pathways activated in the most abundant GM members. Here, using fecal samples obtained from a rat model, we investigated the variation of microbial protein expression in rats subjected to long-term TR feeding. On the other hand, the choice of the fecal sample might be considered as a limit of this study, since fecal microbiota strongly resembles large intestine microbiota, whereas it differs from small intestine microbiota^28^. This sample should be therefore regarded as a reliable proxy for investigating colonic microbial communities; however, considerably different data might be obtained as far as small intestine samples are analyzed.

Differential composition of the GM in TR- vs. AL-fed rats was initially assessed in this study by 16S rRNA gene sequencing. This approach highlighted significant changes in several taxa, including bacteria that are well-known members of the mucosa-associated microbiota (MAM): *Akkermansia*, *Bacteroides* (i.e., *B. rodentium* and *B. thetaiotamicron*), *Bilophila*, *Marvinbryantia*, and *Ruminiclostridium*^29,30^. Two species, *A. muciniphila* and *B. rodentium*, showed the highest relative abundance fold changes (in absolute terms) when comparing TR- and AL-fed rats. Noteworthy, the increased abundance of *A. muciniphila* in TR-fed rats (log_2_FC = 7.3; FDR = 0.0001) is consistent with its mucin-degrading capacity, outperforming other members of the MAM in growth and replication during fasting periods, when mucins are the sole source of nutrients^31^; on the other hand, *B. rodentium*, as well as *B. thetaiotamicron*, is a glycan generalist and mucus degrader and its growth and replication might be fueled more efficiently when a more complex and abundant assortment of diet-derived glycans are available at the mucosa surface for longer periods, as expected in AL-fed rats (log_2_FC = −5.46; FDR < 0.000001)^32,33^.

*A. muciniphila* increased abundance in TR-fed rats is of particular interest. This species is considered a keystone member of the MAM, affecting metabolisms of other commensals^34^. Further, *A. muciniphila* is one of the most relevant species reported to be involved in the complex relationships between diet and metabolic/inflammatory diseases^35–38^. Long-term, high-calorie diets have been already demonstrated to promote the reduction of *Akkermansia* relative abundance and the increase of pathobiont *Bilophila*^39^. An opposite trend by *Akkermansia* and *Bilophila* was observed also in our TR-feeding rat model, suggesting that, while TR feeding might protect against metabolic diseases^6,8,11,16^, its impact on the GM composition might recapitulate that obtained with a balanced host metabolic state.

Our metaproteomic analysis showed that TR feeding is able to induce changes in GM metabolic pathways. Most of the differential functions identified in this study were expressed by members of the mucus colonizers *Akkermansia* and *Lactobacillus*. TR-feeding increased the relative abundance of a specific set of *Akkermansia*-specific proteins, including several metabolic enzymes, such as succinyl-CoA synthetase, phosphoenolpyruvate carboxykinase, N-acetylglucosamine-6-phosphate deacetylase, and protein deglycase. Although no enzymes directly and explicitly involved in mucin degradation could be detected (possibly due to an abundance level lower than the detection limit of the analytic pipeline used), N-acetylglucosamine-6-phosphate deacetylase is known to be implicated in reactions linking mucin degradation to glycolytic/gluconeogenetic pathways, as well as to peptidoglycan biosynthesis^40^. In addition, some of the differential enzymatic functions were not even detectable in the GM of AL-fed rats, suggesting that they might be tightly regulated when the influx of dietary compounds occurs in continuous. Strikingly, the differential abundance of the DNA-binding protein HU-beta might be part of this control in TR-fed rats, given its role in active DNA metabolic transactions^41,42^. This protein stabilizes DNA conformations required to promote and sustain the regulation of many enzymes involved in energy metabolism and catabolism pathways^43^. We hypothesize that the growth of *A. muciniphila* in TR-fed rats may be sustained prevalently by mucin, leading to a blooming of this species. *A. muciniphila* might exploit the advantage of continuous availability of mucin as a nutritional source by a tight (HU protein-dependent) control over the expression of metabolic unnecessary functions, in response to the energy requirement in the fasting gut environment. Furthermore, according to the recently reported ecological co-exclusion behavior of the *Akkermansia* species^44^, we believe that the TR feeding-dependent increase of *Akkermansia* genomic copies and protein functions, observed side by side in the present study, are referred uniquely to strains belonging to *A. muciniphila*.

A further interesting finding of our metaproteomic analyses was the increased abundance of functions expressed by *L. reuteri* and, overall, by the *Lactobacillus*/Lactobacillaceae taxa in the fecal microbiota of TR-fed rats. However, in this case, sequencing analyses showed no differential distribution of *Lactobacillus* species between the two groups of rats. The lack of consensus between 16S rRNA gene sequencing and metaproteomics GM data has already been widely acknowledged as probably due to the different methods of molecular target extraction, differences in the sequences database chosen, and amplification/identification biases^45–47^. Nonetheless, previous DNA sequencing studies reported an increase in the relative proportion of *Lactobacillus* spp. in the GM of rodents subjected to CR or IF^26–30^. Probably, the 16S rRNA gene sequencing pipeline used in this study was less sensitive than the metaproteomic pipeline and did not allow us to confirm previous reports. Lactobacilli are the most numerous and diverse group among lactic acid bacteria that inhabit mucosal surfaces of the oral cavity and the gastrointestinal tract in many animal species, including humans and rodents^27,48^. Lactobacilli are also known to adhere to the intestinal surface through the interaction with mucins, as well as to be able to induce mucin production^49^. In addition, lactobacilli possess inducible proteolytic activity enabling digestion of mucin backbones, and their relative abundance was found to be significantly decreased in *Muc2*^−/−^ mice^50^. Consistently, sequencing of numerous *Lactobacillus* genomes has revealed a wide assortment of genes encoding glycoside hydrolases active on carbohydrate moieties of host mucins and oligosaccharides^51^. Of note, the presence of mucin-degrading bacteria in the GM, when combined with a fiber-rich diet, has been associated to an increased gut health^52–54^. This might lead to hypothesize a link between TR feeding, mucin degradation, and intestinal homeostasis. In addition to the *A. muciniphila*-specific differential functions described above, we also observed that several *Lactobacillus*-specific enzymes are involved in the metabolism of galactose (likely, in the degradation of galactose-containing glycans) and taxonomically assigned to lactobacilli were clearly more abundant in the animals subjected to TR diet. Since TR- and AL-fed rats were administered the same (low-fat) diet, we can speculate that the higher expression by lactobacilli of enzymes involved in galactose metabolism in the GM of fasted rats might be induced by a relatively increased access to the main endogenous source of galactose, namely, gut mucins. Furthermore, monosaccharide and oligopeptide transporters were found as significantly more expressed by *Lactobacillus* spp. colonizing TR-fed rats, possibly related to the uptake of carbohydrates and peptides originated from catabolism. Other differential *Lactobacillus*-specific functions identified in this study can shed light on further biological mechanisms associated to TR feeding. Cell division protein FtsZ resulted among the *Lactobacillus*-specific functions more abundant in the GM of TR-fed rats compared to AL-fed rats. Given its rapid degradation, the dynamics of FtsZ concentration have been elegantly shown to predict cell division rate, particularly in environments where non-dividing microbes receive nutrients in small quantities, such as in the gut^55^. Therefore, the higher abundance of cell division protein FtsZ might account for an increased rate of *Lactobacillus* replication in TR-fed rats. Another of the enzymes varying their abundance in TR-fed animals was arabinose isomerase, which catalyzes the conversion of l-arabinose (usually produced by other microbes able to degrade plant polysaccharides) to l-ribulose, confirming the existence of cross-feeding mechanisms. Interestingly, previous studies reported the ability of some *Lactobacillus* strains to secrete this enzyme, causing an anti-hyperglycemic effect in mice^56^.

Besides *Akkermansia* and *Lactobacillus*, this study reports other (less abundant and less known) GM members as possibly influenced by TR feeding. Namely, *Faecalibaculum* abundance was significantly increased in TR-fed rats according to metaproteomic data. Previously, in a mouse model of Alzheimer’s disease, *Faecalibaculum* was found as more abundant in CR-fed animals compared to AL-fed ones; moreover, in the same animals, both *Faecalibaculum* and *Lactobacillus* have been strongly associated with a protective effect, i.e., a reduction of amyloid-beta plaque deposition^57^. As for *Lactobacillus* and *A. muciniphila*, TR-dependent changes in the gut mucosal ecosystem (i.e., the extended availability of host mucus as the main source of nutrients) might be one of the main causes of the relatively increased abundance of *Faecalibaculum*. In a recent study, *Faecalibaculum rodentium* has been found to be strongly depleted during tumorigenesis and to be able to reduce tumor growth through the production of short-chain fatty acids^58^; intriguingly, its depletion occurs together with mucus changes. Expansion of *Faecalibaculum* in TR-treated rats might thus occur as this genus belongs to GM members whose survival and growth appear to be warranted by availability of host mucus, rather than of substrates from the diet. Prevotellaceae were also found to increase in the TR-fed group of rats according to 16S rRNA gene sequencing results. This finding is consistent with the recent observation of Wang et al. in swine, where TR feeding-dependent increase of Prevotellaceae abundance was negatively correlated to 2-amino-butyrate, a metabolite previously associated with high cardiovascular risk^59^. Finally, beta-lactamase encoded by *Candidatus Arthromitus* was detected in higher abundance in AL-fed than in TR-fed rats. While antibiotic-resistant genes, as part of the GM resistome, have been detected in subjects with no history of antibiotic treatment, their abundance is reported to increase in individuals (obese and overweight) with increased calorie intake^60,61^.

In conclusion, we observed that TR feeding has a relevant impact on GM functions, according to our rat model. This fasting-associated dietary regimen appears to specifically boost next-generation probiotic *A. muciniphila* and several biological activities exerted by other GM members (in particular, proteolytic and galactose metabolism enzymes expressed by *Lactobacillus* spp.). As mucin-degrading activity has been often related to gut homeostasis, a mechanism linking TR feeding, an increase of beneficial *A. muciniphila* and *Lactobacillus* protein functions, degradation of mucin, and intestinal health can be hypothesized. Further investigations integrating DNA sequencing, metaproteomics, and metabolomic analyses, as well as mechanistic and functional studies, are needed to confirm this hypothesis and to deepen our knowledge about molecular aspects of diet-induced changes in the host-microbiota relationship.

## Methods

### Animal intervention and sampling

This study has been performed with a colony of DPP-IV—Fischer 344 male rats bred in-house at the Department of Biomedical Sciences, University of Cagliari. Rats were maintained on an alternating 12-h light/dark cycle (light on at 7 p.m., light off at 7 a.m.), in a temperature- and humidity-controlled environment, with water available AL, and housed two for each cage. Animals were fed AL until the age of 8 weeks with Purina Rodent Lab Chow 4RF21 diet (percentage composition: water 12%, protein 18.5%, fat 3%, fiber 6%, ash 7%, nitrogen-free extract 53.5%; Mucedola srl, Settimo Milanese, Italy). After 8 weeks, rats were divided into 2 groups (each of 8 animals, with 2 rats per cage): the AL control group and the TR feeding group. More specifically, the AL group had AL access to food during both light and dark phases, while the TR group had AL access to food for 8 h during the dark phase, namely from 11 a.m. to 7 p.m. (4 h after lights off). Fecal samples were collected after 48 weeks of diet regimen. Rats were individually placed in a separate cage for fecal sample collection and feces were immediately harvested and stored at −80 °C. Rats received humane care according to the criteria outlined in the National Institutes of Health Publication 86-23, revised 1985. Animal studies were reviewed and approved by the Institutional Animal Care and Use Committee of the University of Cagliari and were performed in accordance with the relevant ethical guidelines and regulations (authorization of the Italian Health Ministry No. 840/2016-PR).

Fecal samples (*N* = 16) were immediately stored at −80 °C until use. At the time of the analyses, stool samples were thawed at 4 °C and divided into two portions, for DNA and protein extraction, respectively.

### DNA extraction and 16S rRNA gene sequencing

Sixteen fecal samples, collected from rats belonging to AL and TR groups, were subjected to DNA extraction with the QIAamp Fast DNA Stool Mini Kit (Qiagen, Hilden, Germany). The extracted DNA was purified according to E.Z.N.A.^®^ Soil DNA Kit (Omega Bio-Tek, Norcross, GA, USA). DNA quality and yield were evaluated via agarose gel and Qubit^TM^ Fluorometer (Life Technologies, Carlsbad, CA, USA, now Thermo Fisher Scientific). Libraries were constructed using Illumina’s recommendations as implemented in the 16S Metagenomic Sequencing Library Preparation guide.

The variable region 4 (V4) of the gene encoding the 16S rRNA was amplified using the 515F and 806R primers (GTGCCAGCMGCCGCGGTAA and GGACTACHVGGGTWTCTAAT, respectively), modified to contain adaptors for MiSeq sequencing (Illumina, San Diego, CA, USA). Two separate gene amplification reactions were performed for each sample, and the products were pooled together and cleaned up using Agencourt AMPure XP Beads (Beckman Coulter Genomics, MA, USA). The next PCR attached dual index barcodes using the Illumina Nextera XT kit so that the PCR products may be pooled and sequenced directly. The final quality control and quantification were conducted using the BioAnalyzer 2100 instrument (Agilent Technologies, Santa Clara, CA, USA). Libraries (average size 440 bps) were quantified with the Qubit^TM^ Fluorometer, normalized, and then pooled equimolar, including 10% PhiX as an internal control. DNA sequencing was performed on the Illumina MiSeq platform, using v3 chemistry (following the manufacturer’s specifications), to generate paired-end reads of 201 bases in length in each direction.

### Analysis of 16S rRNA gene sequencing data

Primers spanning the V4 region (515F and 806R) were removed from the generated fastq files by cutadapt^62^. Reads were further analyzed with the Quantitative Insights Into Microbial Ecology 2 (QIIME2) pipeline (v.2-2021.2)^63^. Initially, DADA2^64^ was used to inspect reads quality and create sequencing error profiles, truncate (both forward and reverse reads to 180 bp), assemble read pairs, remove chimeras, and infer the ASVs present. Taxonomy was assigned using pre-formatted SILVA 138 SSURef NR99^65^ full-length reference sequence and taxonomy files, processed using the RESCRIPt plugin and q2-feature-classifier^66^, provided in the QIIME2 data resources (https://docs.qiime2.org/2021.2/data-resources). A supervised classification method was applied to classify the representative sequences from our V4 dataset, training the Naïve Bayes classifier^67^ using SILVA 138 reference sequences.

Pre-processing of the ASV table was performed using phyloseq^68^ package (v.1.28.0) in R (v3.6.3; https://www.R-project.org). Filtering was done by removing ASVs classified as chloroplast, mitochondria, or without kingdom-level classification from sequencing data. In addition, taxa with unconventional nomenclature were manually filtered out for the differential analysis. The taxa for which a binomial nomenclature was not available are marked in red in Supplementary Data 1.

### Protein extraction and digestion

Sixteen fecal samples were subjected to an established protocol for protein extraction from stool^69^. Specifically, fecal samples (mean weight 102 mg) were resuspended in extraction buffer (2% sodium dodecyl sulfate, 100 mM DTT, 20 mM Tris-HCl pH 8.8), adding 250 μl of buffer per 100 mg of feces. Samples were incubated at 95 °C for 20 min in agitation (500 rpm) in a Thermomixer Comfort (Eppendorf, Hamburg, Germany) and then subjected to bead beating as follows, after adding a steel bead (5 mm diameter; Qiagen) to each sample. Sequentially, samples were: incubated at −80 °C for 10 min; subjected to bead beating for 10 min (30 cycles/s in a TissueLyser LT mechanical homogenizer, Qiagen); incubated at −80 °C for 10 min; incubated at 95 °C for 10 min; subjected to bead beating for 10 min (30 cycles/s); centrifuged at 20,000 × *g* for 10 min at 4 °C. The final supernatant was collected as the fecal protein extract.

Protein extracts were processed according to a modified FASP protocol^70–72^. Twenty microlitres of each protein extract were diluted to 400 μl with UA solution (8 M urea in 100 mM Tris-HCl, pH 8.8), filtered using an Ultrafree-MC centrifugal filter (0.22 µm; Merck Millipore, Billerica, MA, USA), according to manufacturers’ instructions, and loaded onto an Amicon Ultra-0.5 (cutoff 10 kDa) filtration device (Merck Millipore) and centrifuged at 14,000 × *g* for 15 min. Then, sequentially, each sample was subjected to addition of 200 μl of UA solution and centrifugation (as described above); addition of 100 μl of 50 mM iodoacetamide in UA solution, incubation at RT for 20 min, and centrifugation; addition of 100 μl of UA solution and centrifugation (twice); addition of 100 μl of 50 mM ammonium bicarbonate and centrifugation (twice); addition of 100 μl of trypsin solution (1 μg in 50 mM ammonium bicarbonate) and incubation at 37 °C overnight. Peptide mixtures were collected by centrifugation, followed by an additional elution with 100 μl of a 20% acetonitrile, 0.2% formic acid solution. Finally, peptide mixtures were brought to dryness and reconstituted in 0.2% formic acid. Peptide mixtures concentration was estimated by measuring absorbance at 280 nm with a NanoDrop 2000 spectrophotometer (Thermo Fisher Scientific, Waltham, MS, USA), using dilutions of the MassPREP *E. coli* Digest Standard (Waters, Milford, MA, USA) to generate a calibration curve.

### Liquid chromatography-mass spectrometry analysis

Liquid chromatography (LC)–tandem mass spectrometry (MS/MS) analyses were performed on an LTQ Orbitrap Velos mass spectrometer (Thermo Fisher Scientific), operating with an EASY-spray source, interfaced with an UltiMate 3000 RSLCnano LC system (Thermo Fisher Scientific). Each sample was run once and analyses were run in a randomized order.

Peptide mixtures (4 μg per run) were loaded, concentrated, and desalted on a trapping pre-column (Acclaim PepMap C18, 75 μm × 2 cm nanoViper, 3 μm, 100 Å, Thermo Fisher Scientific), using 0.2% formic acid at a flow rate of 5 μl/min. The peptide separation was performed with a C18 EASY-spray column (PepMap RSLC C18, 75 μm × 50 cm, 2 μm, 100 Å, Thermo Fisher Scientific) at 35 °C with a flow rate of 250 nL/min for 247 min, using the following two-step gradient of eluent B (0.2% formic acid in 95% ACN) in eluent A (0.2% formic acid in 5% ACN): 2.5–37.5% for 242 min and 37.5–99% for 5 min.

The mass spectrometer was set up in a data-dependent MS/MS mode, where a full scan spectrum (from 375 to 2000 m/z) is followed by MS/MS spectra, under the direct control of the Xcalibur software (v.2.2 SP1). The instrument operated in positive mode. The temperature of the ion transfer capillary and the spray voltage were set to 250 °C and 1.85 kV, respectively. Full scans and MS/MS spectra were acquired in the Orbitrap with resolutions of 60,000 and 7500 at 400 m/z, respectively. The automatic gain control was set to 1,000,000 ions, and the lock mass option was enabled on a protonated polydimethylcyclosiloxane background ion as an internal recalibration for accurate mass measurements. Peptide ions were selected as the 10 most intense peaks of the previous scan; the signal threshold for triggering an MS/MS event was set to 500 counts, and dynamic exclusion was set to 30 s. Higher-energy collisional dissociation was used as the fragmentation method, by applying a 35% value for normalized collision energy, an isolation width of m/z 3.0, a *Q*-value of 0.25, and an activation time of 0.1 ms. Nitrogen was used as the collision gas.

### Peptide identification and annotation

Peptide identification was carried out using the Proteome Discoverer informatics platform (v.2.4.1.15; Thermo Fisher Scientific), with Sequest-HT as a search engine and Percolator for peptide validation (FDR < 1%). Search parameters were set as follows: precursor mass threshold 350–5000 Da; minimum peak count 6; signal-to-noise threshold 2; enzyme trypsin; maximum missed cleavage sites 2; peptide length range 5–50 amino acids; precursor mass tolerance 10 ppm; fragment mass tolerance 0.02 Da; dynamic modification methionine oxidation; static modification cysteine carbamidomethylation.

Quantification was carried out using the Proteome Discoverer nodes “Minora Feature Detector”, “Feature Mapper”, and “Precursor Ions Quantifier”. The integrated peak area of the most abundant peak at the apex of the chromatographic profile was used as a quantitative measure, after being subjected to a normalization step based on the total peptide intensity of the samples, based on the results of a recent comparison among MS1-based label-free protein quantification tools^73^.

The identification node was built on a combination of databases. Two microbial sequence databases were used: (i) a collection of metagenomic sequences obtained in house from rat fecal samples (13,163,507 sequences; file name: DB1.fasta); (ii) a publicly available rat metagenomic dataset (ftp://ftp.cngb.org/pub/SciRAID/Microbiome/rat/GeneCatalog/rat_geneset.pep.gz; 5,130,167 sequences; file name: DB2.fasta)^74^. Moreover, two additional databases, containing host (reference proteome for *Rattus norvegicus*; https://www.uniprot.org/proteomes/UP000002494 release 2021_01; 29,936 sequences; file name: DB3.fasta) and food (reference proteome for *Glycine max*; https://www.uniprot.org/proteomes/UP000008827 release 2020_12; 74,863 sequences; file name: DB4.fasta) protein sequences, were employed.

The file named PeptideGroups.txt (available in the ProteomeXchange repository, see “Data availability statement” for details) was used as input for statistical analyses.

Taxonomic annotation was obtained according to the following steps: first, protein sequences were subjected to a DIAMOND (v.0.8.22) search against the NCBI-nr database (2021/05 update), using the blastp command with default parameters^75^; then, DIAMOND outputs were loaded on MEGAN (v.6.19.9)^76^ using default parameters. Only species annotated with binomial nomenclature were considered for differential analysis; the species for which a binomial nomenclature was not available are marked in red in Supplementary Data 3.

Functional characterization allowed the classification of protein sequences according to UniProt protein families and metabolic pathways^77^, COG of proteins^78^, KOG^79^, and CAZy^80^.

Protein families and metabolic pathways were obtained through the following steps: first, the identified protein sequences were aligned against a database containing all bacterial sequences from UniProtKB/Swiss-Prot (release 2021_05) using DIAMOND (blastp module, *e*-value threshold 10^−5^); then, UniProtKB/Swiss-Prot accession numbers were exploited to retrieve specific information from the UniProt website (https://www.uniprot.org) via the “retrieve” tool. COG, KOG, and CAZy information was achieved using the eggnog-mapper package (v.2.0.1)^81^ available in a Galaxy server (https://proteomics.usegalaxy.eu)^82^, according to the following parameters: eggnog database v.2.0; min e-value threshold 0.001; min bit score threshold 60. In the case of the assignment of a single peptide sequence to multiple protein entries, the first protein entry in the list having a valid blastp output was selected.

### Statistical analysis and graph generation

PCoA plots were generated using the MicrobiomeAnalyst web application (https://www.microbiomeanalyst.ca), which also calculates the statistical significance of group clustering through a PERMANOVA test^83^.

Differential analysis of 16S rRNA gene sequencing data was performed on count data at various taxonomic levels, obtained by aggregating ASVs based on their taxonomy assignment, through the MicrobiomeAnalyst web application. Features with valid values in less than six samples in at least one of the compared groups were filtered out. Count data were transformed prior to statistical testing according to the Relative Log Expression method^84^. Differential abundance analysis was then carried out using the edgeR algorithm^85^. The *p*-value correction for multiple tests was performed by calculating an FDR^86^, and results were considered as significant for FDR < 0.1.

Differential analysis of metaproteomic data was performed using the Perseus computational platform (v.1.6.15.0)^87^, using as inputs peptide area values (aggregated based on the functional and taxonomic annotation levels), according to the following steps: (i) data log-transformation: abundance data were subjected to binary logarithmic transformation to approximate a normal distribution, subsequently verified using the Shapiro–Wilk test; (ii) protein filtering: features not reaching 75% valid values in at least one group were filtered out; (iii) missing value (MV) replacement: MVs were replaced with a constant value, calculated for each comparison as the binary logarithm of the minimum of the distribution (approximated to the nearest integer) minus 1; (iv) differential analysis: differential protein abundances between groups were tested with a two-tail Student’s *t* test; (v) correction for multiple testing: FDR was calculated based on permutation-adjustment considering *q* = 0.1 as the threshold of significance. An abundance log-ratio (LR) was also computed using Microsoft Excel (Redmond, WA, USA) as a quantitative measure of the change in abundance of a feature between the two sample groups. The LR was calculated for each feature, starting from its original abundance data, as the binary logarithm of the ratio between the mean abundances measured in the two sample groups (after summing a background correction factor equal to 1000 to the mean abundances) for that feature.

Heatmaps were generated using the web application Morpheus (https://software.broadinstitute.org/morpheus/). The galactose metabolism pathway was reconstructed based on the corresponding KEGG pathway map, available at http://www.genome.jp/kegg/pathway.html^79^, and further refined based on recently reviewed data concerning galactose metabolism in lactic bacteria^88^.

### Reporting summary

Further information on research design is available in the Nature Research Reporting Summary linked to this article.

## Supplementary information

Supplementary Information

Supplementary Data 1

Supplementary Data 2

Supplementary Data 3

Supplementary Data 4

Supplementary Data 5

Supplementary Data 6

Supplementary Data 7

Supplementary Data 8

Supplementary Data 9

Reporting Summary

## Data Availability

The mass spectrometry proteomics data (including all sequence databases) have been deposited to the ProteomeXchange Consortium via the PRIDE^89^ partner repository with the dataset identifier PXD024509. The 16S rRNA gene sequencing data have been deposited to the European Nucleotide Archive (ENA) with the identifier PRJEB46472.
