# Supplementary material for: Time-restricted feeding induces Lactobacillus- and Akkermansia-specific functional changes in the rat fecal microbiota
Source: NPJ Biofilms Microbiomes. 2021 Dec 3;7:85. doi: 10.1038/s41522-021-00256-x (PMC8642412; doi:10.1038/s41522-021-00256-x)
Supplement: Supplementary file 1 — Supplementary Information [file 41522_2021_256_MOESM1_ESM.pdf]

Supplementary Information

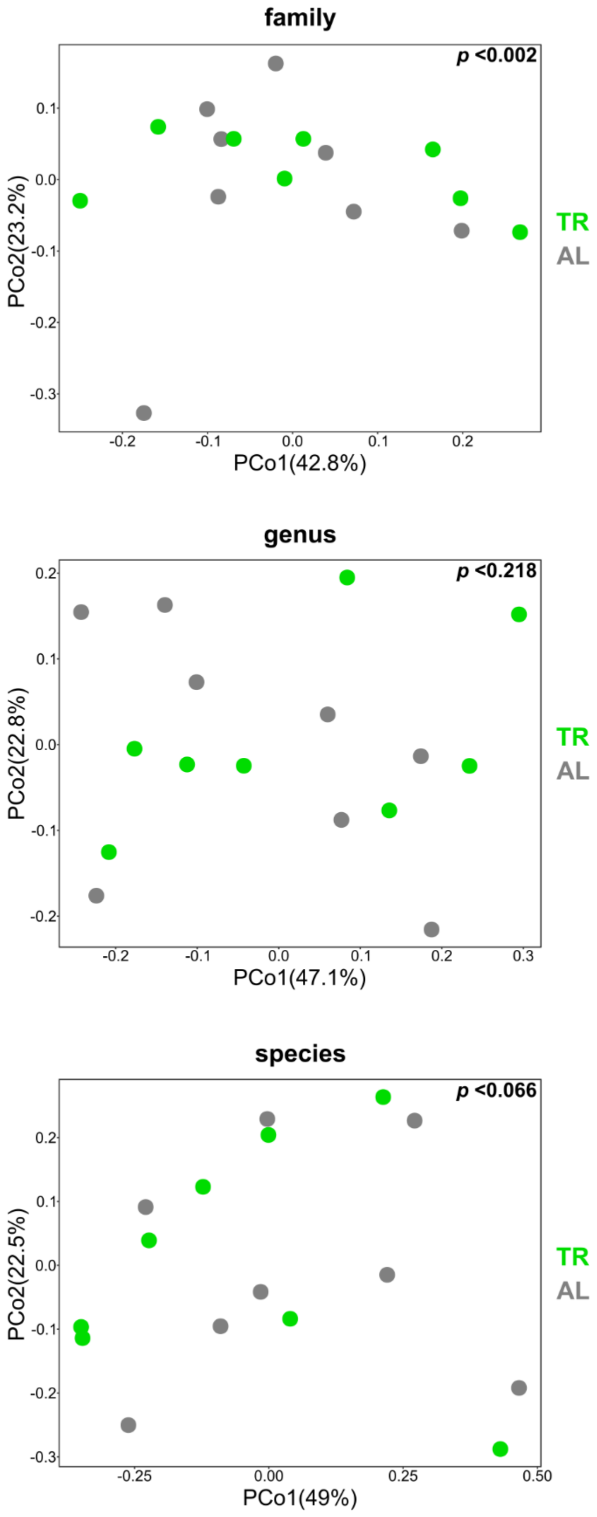

Supplementary Figure 1 Beta-diversity between TR- and AL-fed rats according to 16S rRNA gene sequencing results. PCoA plot based on microbial family (top), genus (middle), and species (bottom) abundance data. Each dot indicates a different sample. PERMANOVA p-values are also shown.

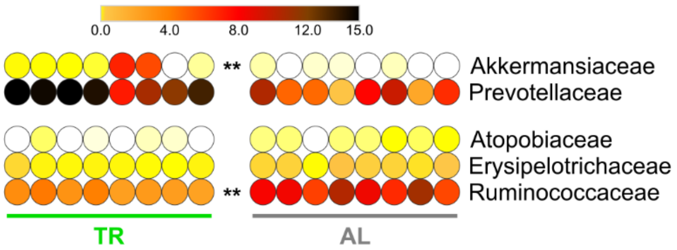

Supplementary Figure 2 Changes in taxonomic family composition in the fecal microbiota of TR- *vs* AL-fed rats according to 16S rRNA gene sequencing results. Heatmap illustrating microbial genera with significantly differential abundance between AL and TR groups (FDR<0.1). A double asterisk refers to FDR <0.001. Each dot indicates a different sample. The color gradient is based on the relative abundance of the family.

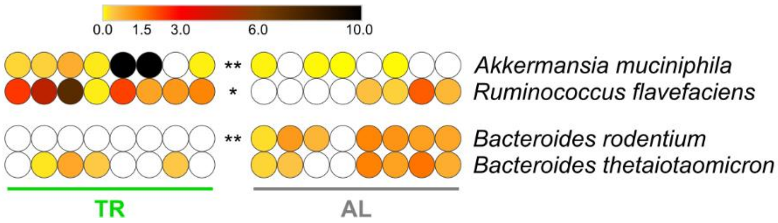

Supplementary Figure 3 Changes in taxonomic species composition in the fecal microbiota of TR- *vs* AL-fed rats according to 16S rRNA gene sequencing results. Heatmap illustrating microbial genera with significantly differential abundance between AL and TR groups (FDR<0.1). A single or double asterisk refers to FDR<0.01 or <0.001, respectively. Each dot indicates a different sample. The color gradient is based on the relative abundance of the species.

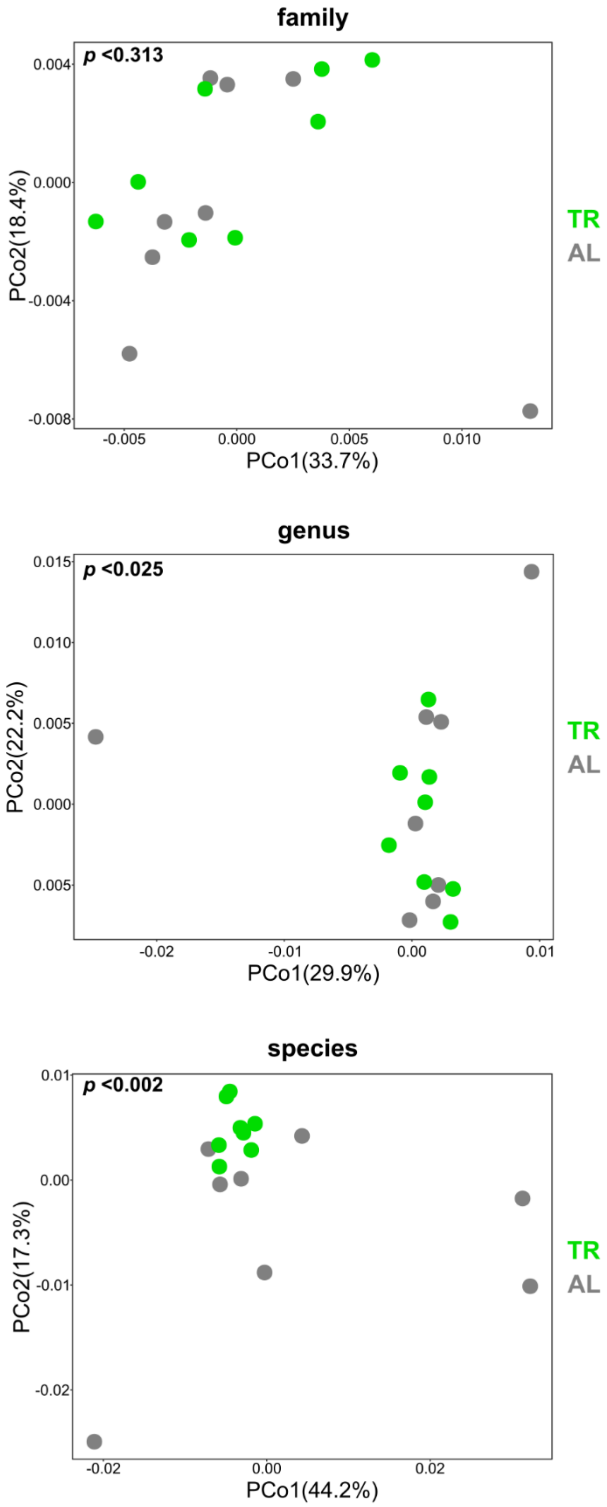

Supplementary Figure 4 Beta-diversity between TR- and AL-fed rats according to metaproteomic taxonomic results. PCoA plot based on microbial family (top), genus (middle), and species (bottom) abundance data. Each dot indicates a different sample. PERMANOVA p-values are also shown.

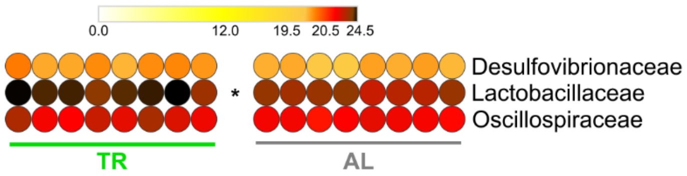

Supplementary Figure 5 Changes in taxonomic family composition in the fecal metaproteome of TR- *vs* AL-fed rats. Heatmap illustrating microbial genera with significantly differential abundance between AL and TR groups (FDR<0.1). A single asterisk refers to FDR<0.01. Each dot indicates a different sample. The color gradient is based on the relative abundance of the family.

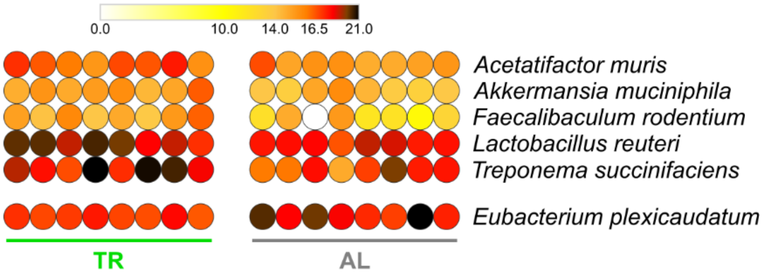

Supplementary Figure 6 Changes in taxonomic species composition in the fecal metaproteome of TR- *vs* AL-fed rats. Heatmap illustrating microbial genera with significantly differential abundance between AL and TR groups (FDR<0.1). Each dot indicates a different sample. The color gradient is based on the relative abundance of the species.

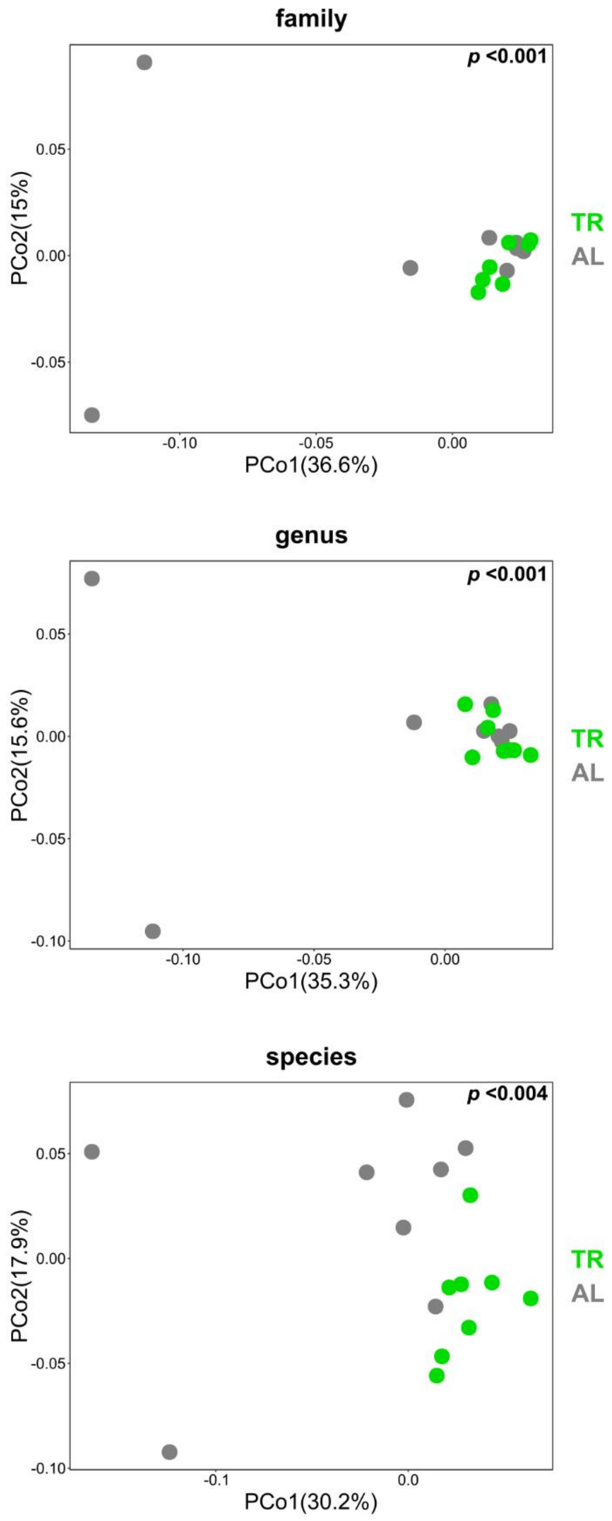

Supplementary Figure 7 Beta-diversity between TR- and AL-fed rats according to taxa-specific functional results. PCoA plot based on microbial family-specific (top), genus-specific (middle), and species-specific (bottom) function abundance data. Each dot indicates a different sample. PERMANOVA p-values are also shown.

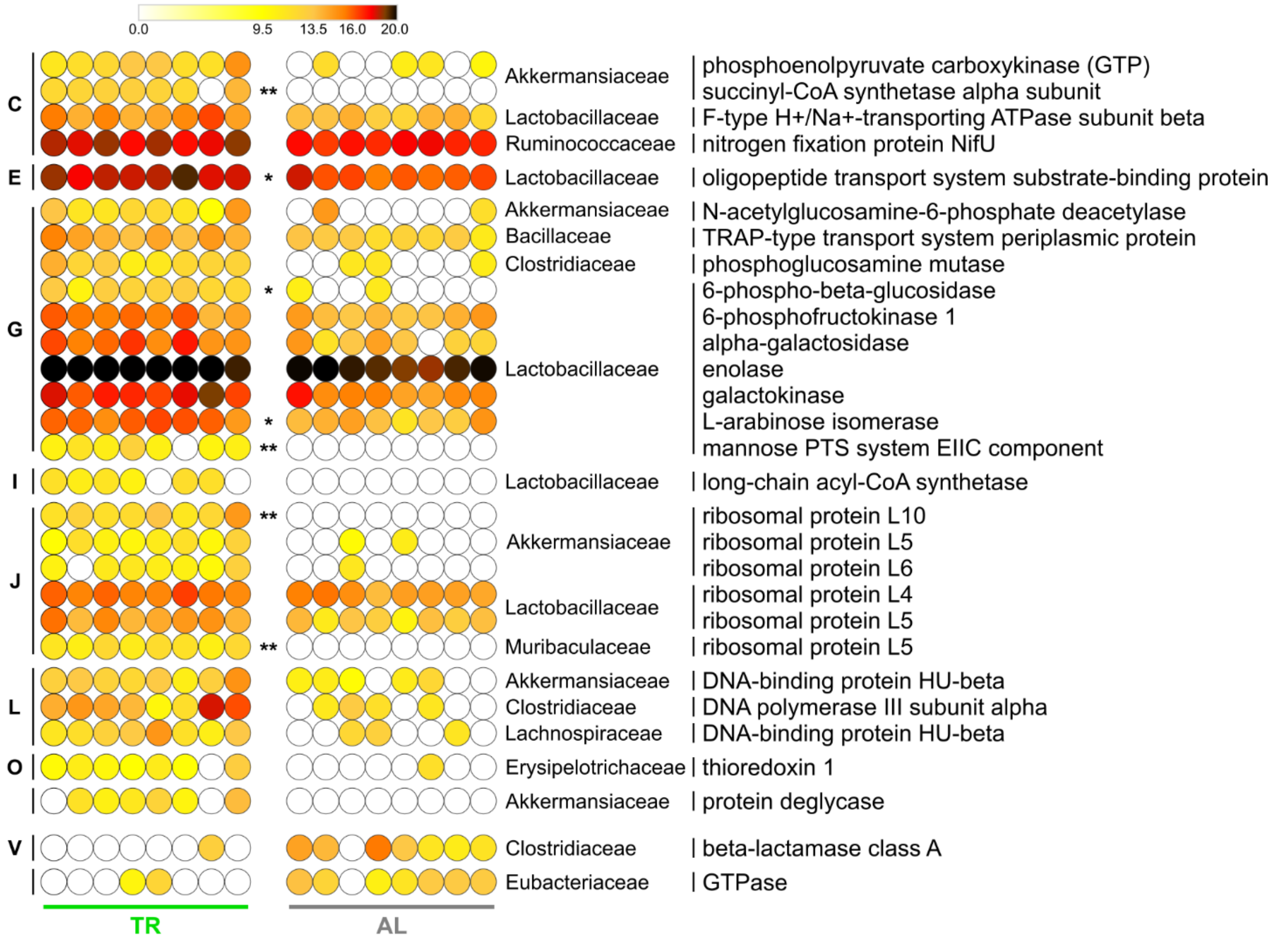

Supplementary Figure 8 Changes in the functional profile of the fecal metaproteome of TR- *vs* AL-fed rats. Heatmap illustrating family-specific functions with significantly differential abundance between AL and TR groups (FDR<0.1). A single or double asterisk refers to FDR<0.01 or <0.001, respectively. Each dot indicates a different sample. The color gradient is based on the relative abundance of functions. Functions are ordered sequentially: i) according to the group of rats in which they are significantly more abundant, indicated in the bottom; ii) according to their COG category, indicated close to the left margin of the heatmap (C, Energy production and conversion; E, Amino acid transport and metabolism; G, Carbohydrate transport and metabolism; I, Lipid transport and metabolism; J, Translation, ribosomal structure and biogenesis; L, Replication, recombination and repair; O, Posttranslational modification, protein turnover, chaperones; V, Defense mechanisms); iii) according to the taxonomic family to which they were assigned, indicated close to the right margin of the heatmap; iv) in alphabetical order.

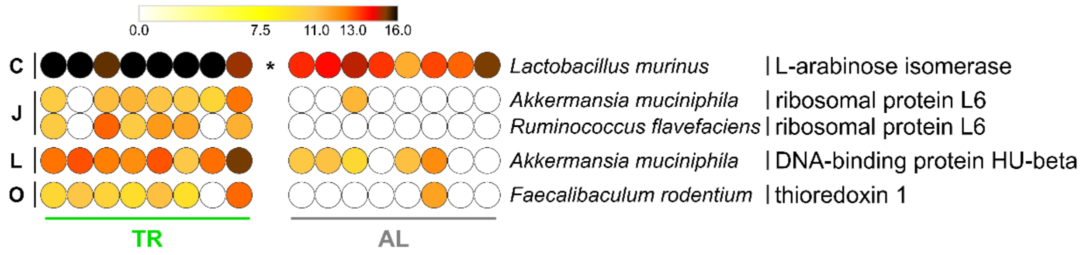

Supplementary Figure 9 Changes in the functional profile of the fecal metaproteome of TR- *vs* AL-fed rats. Heatmap illustrating species-specific functions with significantly differential abundance between AL and TR groups (FDR<0.1). A single asterisk refers to FDR<0.01. Each dot indicates a different sample. The color gradient is based on the relative abundance of functions. Functions are ordered sequentially: i) according to the group of rats in which they are significantly more abundant, indicated in the bottom; ii) according to their COG category, indicated close to the left margin of the heatmap (C, Energy production and conversion; J, Translation, ribosomal structure and biogenesis; L, Replication, recombination and repair; O, Posttranslational modification, protein turnover, chaperones); iii) according to the taxonomic species to which they were assigned, indicated close to the right margin of the heatmap.

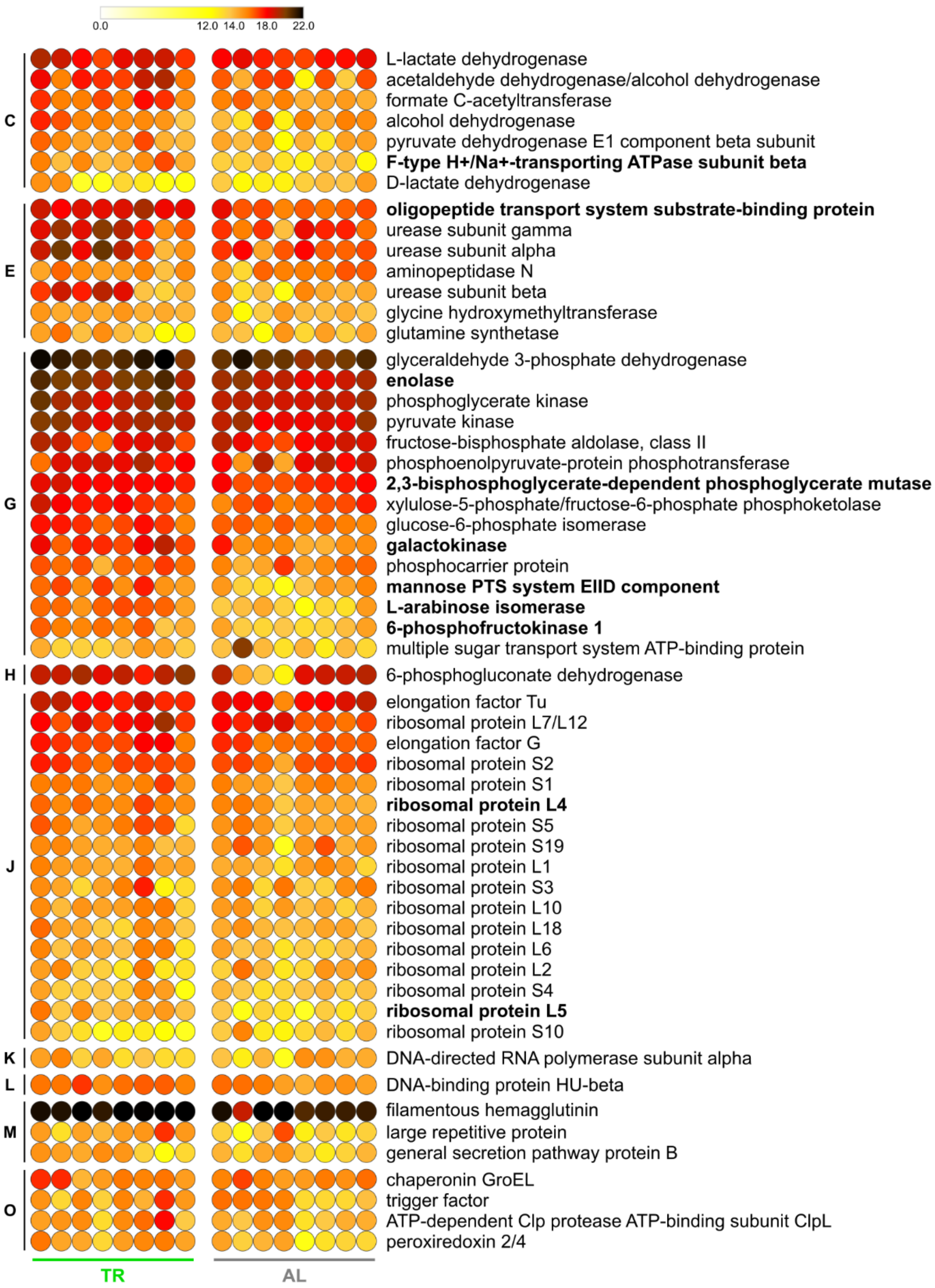

Supplementary Figure 10 ***Lactobacillus*-specific functions detected in all fecal metaproteome samples analyzed in this study.** Functions in bold are differentially abundant between the two groups (FDR<0.1; see Figure 4). Each dot indicates a different sample. The color gradient is based on the relative abundance of functions. Functions are ordered sequentially: i) according to their COG category, indicated close to the left margin of the heatmap (C, Energy production and conversion; E, Amino acid transport and metabolism; G, Carbohydrate transport and metabolism; H, Coenzyme transport and metabolism; J, Translation, ribosomal structure and biogenesis; K, Transcription ; L, Replication, recombination and repair; M, Cell wall/membrane/envelope biogenesis; O, Posttranslational modification, protein turnover, chaperones); ii) according to their average relative abundance.

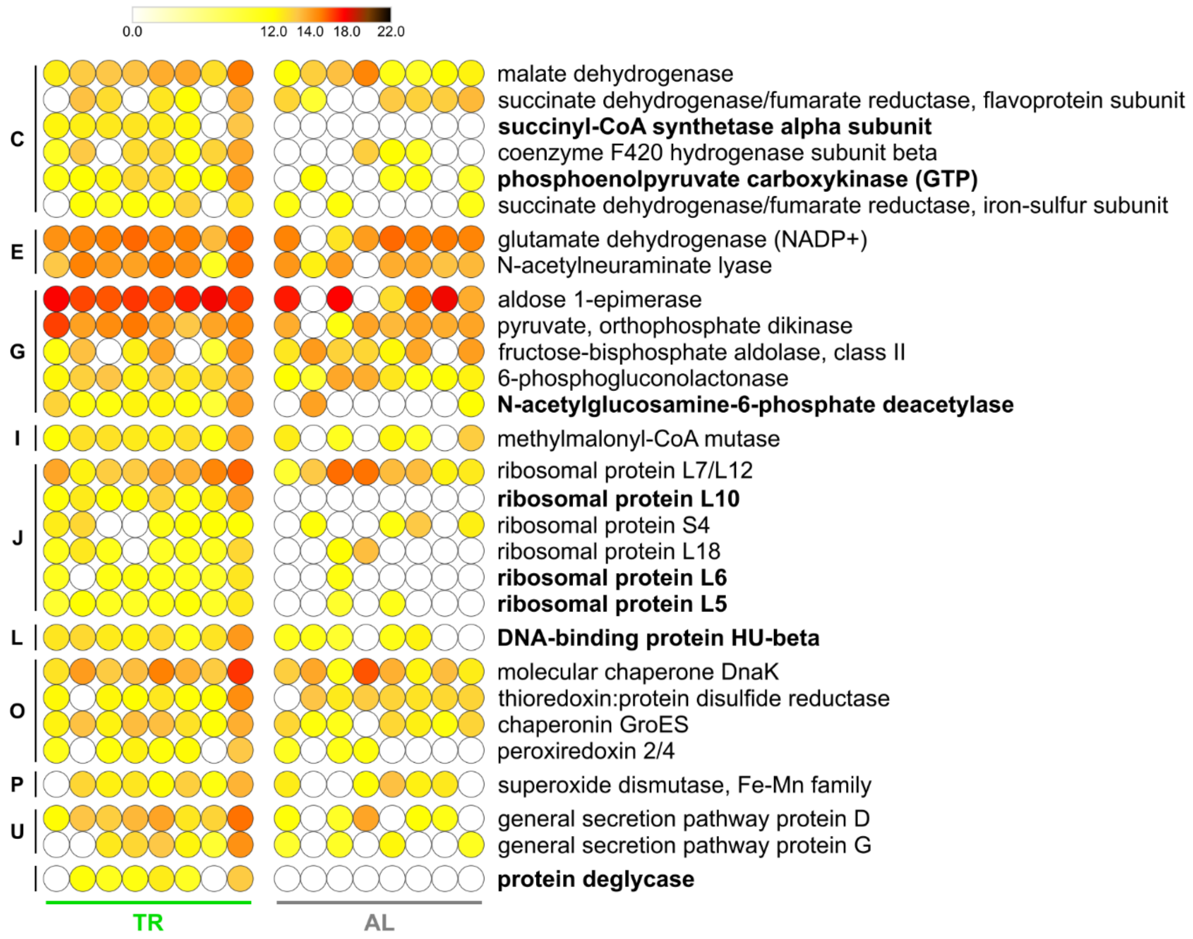

Supplementary Figure 11 ***Akkermansia*-specific functions detected in TR and AL fecal metaproteome samples.** Functions detected in at least 6 samples of at least one group are shown. Functions in bold are differentially abundant between the two groups (FDR<0.1; see Figure 4). Each dot indicates a different sample. The color gradient is based on the relative abundance of functions. Functions are ordered sequentially: i) according to their COG category, indicated close to the left margin of the heatmap (C, Energy production and conversion; E, Amino acid transport and metabolism; G, Carbohydrate transport and metabolism; I, Lipid transport and metabolism; J, Translation, ribosomal structure and biogenesis; L, Replication, recombination and repair; O, Posttranslational modification, protein turnover, chaperones; P, Inorganic ion transport and metabolism; U, Intracellular trafficking, secretion, and vesicular transport); ii) according to their average relative abundance.
