# Supplementary material for: Time-restricted feeding induces Lactobacillus- and Akkermansia-specific functional changes in the rat fecal microbiota
Source: NPJ Biofilms Microbiomes. 2021 Dec 3;7:85. doi: 10.1038/s41522-021-00256-x (PMC8642412; doi:10.1038/s41522-021-00256-x)
Supplement: Supplementary file 11 — Reporting Summary [file 41522_2021_256_MOESM11_ESM.pdf]

nature portfolio

Corresponding author(s): Sergio Uzzau
Last updated by author(s): Aug 23, 2021

Reporting Summary

Nature Portfolio wishes to improve the reproducibility of the work that we publish. This form provides structure for consistency and transparency in reporting. For further information on Nature Portfolio policies, see our Editorial Policies and the Editorial Policy Checklist.

Statistics

For all statistical analyses, confirm that the following items are present in the figure legend, table legend, main text, or Methods section.

n/a	Confirmed	
☐	☒	The exact sample size (*n*) for each experimental group/condition, given as a discrete number and unit of measurement
☐	☒	A statement on whether measurements were taken from distinct samples or whether the same sample was measured repeatedly
☐	☒	The statistical test(s) used AND whether they are one- or two-sided<br>*Only common tests should be described solely by name; describe more complex techniques in the Methods section.*
☒	☐	A description of all covariates tested
☐	☒	A description of any assumptions or corrections, such as tests of normality and adjustment for multiple comparisons
☒	☐	A full description of the statistical parameters including central tendency (e.g. means) or other basic estimates (e.g. regression coefficient) AND variation (e.g. standard deviation) or associated estimates of uncertainty (e.g. confidence intervals)
☐	☒	For null hypothesis testing, the test statistic (e.g. *F*, *t*, *r*) with confidence intervals, effect sizes, degrees of freedom and *P* value noted<br>*Give P values as exact values whenever suitable.*
☒	☐	For Bayesian analysis, information on the choice of priors and Markov chain Monte Carlo settings
☒	☐	For hierarchical and complex designs, identification of the appropriate level for tests and full reporting of outcomes
☒	☐	Estimates of effect sizes (e.g. Cohen's *d*, Pearson's *r*), indicating how they were calculated

Our web collection on statistics for biologists contains articles on many of the points above.

Software and code

Policy information about availability of computer code

Data collection	Mass spectrometry data were collected using the commercial software Xcalibur (version 2.2 SP1).
Data analysis	The following commercial and open source software was used for data analysis in this study: QIIME2 (v.2-2021.2), phyloseq (v.1.28.0), R (v3.6.3), Proteome Discoverer (v.2.4.1.15), DIAMOND (v.0.8.22), MEGAN (v.6.19.9), eggnog-mapper (v.2.0.1), MicrobiomeAnalyst (web app), Perseus (v.1.6.15.0)

For manuscripts utilizing custom algorithms or software that are central to the research but not yet described in published literature, software must be made available to editors and reviewers. We strongly encourage code deposition in a community repository (e.g. GitHub). See the Nature Portfolio guidelines for submitting code & software for further information.

Data

Policy information about availability of data

All manuscripts must include a data availability statement. This statement should provide the following information, where applicable:

- Accession codes, unique identifiers, or web links for publicly available datasets
- A description of any restrictions on data availability
- For clinical datasets or third party data, please ensure that the statement adheres to our policy

The mass spectrometry proteomics data (including all sequence databases) have been deposited to the ProteomeXchange Consortium via the PRIDE partner repository with the dataset identifier PXD024509.
The 16S rRNA gene sequencing data have been deposited to the European Nucleotide Archive (ENA) with the identifier PRJEB46472.

Field-specific reporting

Please select the one below that is the best fit for your research. If you are not sure, read the appropriate sections before making your selection.

☒ Life sciences ☐ Behavioural & social sciences ☐ Ecological, evolutionary & environmental sciences

For a reference copy of the document with all sections, see nature.com/documents/nr-reporting-summary-flat.pdf

Life sciences study design

All studies must disclose on these points even when the disclosure is negative.

Sample size	No formal power calculation was performed prior to this study. The rationale of the experimental design was based on a previous rat fecal metaproteomics study (Abbondio et al., Front Microbiol 2019), where 4 rats per groups were analyzed and a sufficient number of differential features were detected. Here, we decided to double the number of animals analyzed in order to further improve the statistical power of the study.
Data exclusions	No data were excluded from the analyses.
Replication	The reproducibility of the analytical pipeline used in this study were extensively measured and discussed in a previous methodological paper from our group (Tanca et al., Microbiome 2014). In view of the decent method reproducibility expected, we decided not to perform any sample/run replicate in this study.
Randomization	Samples were run in LC-MS/MS in randomized order. Among covariates, cage effect was evaluated in a preliminary analysis and no significant effect was found (data not shown). Therefore, cage was not considered as a covariate in the final statistical analysis.
Blinding	The operators involved in sample preparation and in mass spectrometry analyses were not aware of the sample grouping.

Reporting for specific materials, systems and methods

We require information from authors about some types of materials, experimental systems and methods used in many studies. Here, indicate whether each material, system or method listed is relevant to your study. If you are not sure if a list item applies to your research, read the appropriate section before selecting a response.

Materials & experimental systems

n/a	Involved in the study	
☒	☐	Antibodies
☒	☐	Eukaryotic cell lines
☒	☐	Palaeontology and archaeology
☐	☒	Animals and other organisms
☒	☐	Human research participants
☒	☐	Clinical data
☒	☐	Dual use research of concern

Methods

n/a	Involved in the study	
☒	☐	ChIP-seq
☒	☐	Flow cytometry
☒	☐	MRI-based neuroimaging

Animals and other organisms

Policy information about studies involving animals; ARRIVE guidelines recommended for reporting animal research

Laboratory animals	This study has been performed with a colony of DPP-IV– Fischer 344 male rats. Samples were collected when the rats were 56-week old.
Wild animals	This study did not involve wild animals.
Field-collected samples	No samples were collected from the field.
Ethics oversight	Rats received humane care according to the criteria outlined in the National Institutes of Health Publication 86-23, revised 1985. Animal studies were reviewed and approved by the Institutional Animal Care and Use Committee of the University of Cagliari and were performed in accordance with the relevant guidelines and regulations (authorization of the Italian Health Ministry No. 840/2016-PR).

Note that full information on the approval of the study protocol must also be provided in the manuscript.
